# Photocatalytic treatment of organic pollutants in a synthetic wastewater using UV light and combinations of TiO_2_, H_2_O_2_ and Fe(III)

**DOI:** 10.1371/journal.pone.0216745

**Published:** 2019-05-15

**Authors:** Stavros G. Poulopoulos, Azat Yerkinova, Gaukhar Ulykbanova, Vassilis J. Inglezakis

**Affiliations:** The Environment & Resource Efficiency Cluster (EREC), Environmental Science & Technology Group (ESTg), Chemical Engineering Department, School of Engineering, Nazarbayev University, Astana, Kazakhstan; University of Iowa, UNITED STATES

## Abstract

In this study, the photocatalytic treatment of an organic wastewater with/without phenolic compounds by means of ultraviolet irradiation, titanium dioxide and hydrogen peroxide was examined in an annular photoreactor. Specifically, the effect of initial total carbon concentration, catalyst loading and H_2_O_2_ amount on the removal of total carbon was first examined in the case of a synthetic organic wastewater. The influence of partial carbon substitution by phenol, 2-chlorophenol, 2,4-discholophenol, trichlorophenol, and 4-nitrophenol on total carbon removal and target compounds’ conversion was studied keeping constant the initial organic carbon load. It was shown that the process applied was effective in treating the wastewater for initial total carbon 32 mg L^-1^, 0.5 g L^-1^ TiO_2_, and 66.6 mg L^-1^ H_2_O_2_. Applying UV/TiO_2_ and UV/H_2_O_2_, 58% and 53% total carbon removals were achieved, respectively, but combining TiO_2_ and H_2_O_2_ did not result in a better performance in the case of the synthetic wastewater without any phenolic compounds. In contrast, when a phenolic compound was added, the addition of H_2_O_2_ was beneficial, eliminating the differences observed from one phenolic compound to another. The total carbon removals observed were lower than the corresponding final conversions of the target phenolic compounds. Finally, the electric energy per order values were calculated and found to range in 52–248 kWh/m^3^/order, being dependent from the process applied and the phenolic compound present in the wastewater.

## 1 Introduction

Water pollution is a globally pressing ecological problem and has adverse impacts on both natural ecosystems and human daily life. The growth of industry along with the unprecedent increase in the human population have led to a high demand for water resources, whereas at the same time large amounts of various wastewaters are generated and threaten the quality of these resources. Food and energy security, sustainable development, human and ecosystems health rely on water availability and quality [1]. Therefore, it is of outmost importance to make sure that industrial wastewaters are adequately treated prior to their disposal so as to minimize the impact on the human health and environment [2]. Although by means of conventional methods and especially of biological processes, 80–90% of all pollutants are usually removed [3], it has been found that hazardous organic pollutants may escape these processes [4].

Chlorophenols constitute a group of organic compounds that is widely used in the dye manufacture, petroleum refineries, herbicide production, and in pharmaceutical industry [5]. They can be found in the environment through the release of polluted water from these industries [6]. They are harmful to humans and ecosystems and considered as potential carcinogenics [7]. Moreover, they are known to exhibit a bio-resistant nature [8]. Advanced Oxidation Processes (AOPs) can be utilized to eliminate such toxic organic pollutants in wastewaters by converting them into water and carbon dioxide [9].

Specifically, AOPs are an attractive alternative when bioresistant organic pollutants are to be mineralized in wastewaters. In the oxidation process, highly active hydroxyl radicals are involved, which are generated by a variety of mechanisms [10]. Among them, photochemical and photocatalytic methods constitute sustainable treatment technologies with “zero” waste [11]. Hydrogen peroxide (H_2_O_2_) is commonly used to provide the active hydroxyl radicals, but its use increases the operating cost of the process, whereas the low oxidation rate when compex wastewaters are processed is the main drawback of titanium dioxide use [12]. The employment of photo-Fenton processes or combining H_2_O_2_ with TiO_2_ could be effective alternatives. Regarding the elimination of phenols and chlorophenols in water, these processes have been proved promising [13]. Particularly, AOPs can effectively decompose toxic and bioresistant compounds in water like phenol, 2-chlorophenol and 2,4-dichlorophenol [13–15]. For example, Andreozzi et al. [16] studied the photochemical oxidation of 2,4-dichlorophenol and 3,4-dichlorophenol using homogeneous photocatalysis with Fe(III) under UV-A irradiation. They reported complete removal of both compounds with increased concentrations of iron and proper adjustment of the solution pH.

There is an extensive list of applications of photochemical processes to eliminate toxic or emerging pollutants in water [17–22], but significantly less research has been conducted to degrade these compounds in complex wastewaters or treat effectively complex wastewaters in terms of carbon removal [23,24]. Photochemical reactions take place through complex and variable pathways based on radical mechanisms that are sensitive to experimental parameters, and thus their efficiency can be adversely influenced by the occurrence of rest compounds in a wastewater [25]. As a result, further research is essential to investigate the application of photochemical processes in the case of complex wastewaters.

In this study, UV/TiO_2_, UV/H_2_O_2_, UV/TiO_2_/H_2_O_2_ and UV/TiO_2_/H_2_O_2_/Fe(III) processes were used to treat a synthetic wastewater containing mainly organic load. The UV/TiO_2_/H_2_O_2_ process was also applied to treat the synthetic wastewater containing the following compounds: Phenol, 2-chlorophenol, 2,4-dichlorophenol, 2,4,6-trichlorophenol, and 4-nitrophenol. Finally, electrical energy consumption due to artificial light irradiation was estimated as it is connected directly to the environmental footprint of photochemical processes.

## 2 Materials and methods

### 2.1. Chemicals and wastewater composition

The composition of the stock synthetic wastewater prepared is shown in Table 1. The total carbon content was 1080 mg L^-1^, 95% organic and 5% inorganic. It is a typical non-toxic and biodegradable wastewater that has been used as substrate to examine the effect of the presence of toxic compounds on the efficiency of wastewater treatment technologies [26,27]. Fresh stock solutions were prepared every three days. The following ratios of stock solution volume to water volume were used to achieve the experimental total carbon of 477, 124, 61, and 32 mg L^-1^, respectively, for the UV/TiO_2_ experiments: 110 mL/140 mL, 28 mL/222 mL, 14 mL/236 mL, and 7.5 mL/242.5. Phenol was supplied by Sigma Aldrich. 2-Chlorophenol (≥99% w/w), 2,4,6-Trichlorophenol (≥98% w/w), and 4-Nitrophenol (≥99% w/w) were purchased from Sigma Aldrich. 2,4-Dichlorophenol (≥99% w/w) was supplied by Acros Organics. Titanium (IV) dioxide (Aeroxide P25, nanopowder, 21 nm average primary particle size, ≥99.5% trace metals basis, 80/20 anatase to rutile weight ratio, specific surface area 50 m^2^ g^-1^) from Sigma-Aldrich was used as catalyst, whereas hydrogen peroxide (37.6% w/w) was purchased from Skat-Reactiv. Iron chloride anhydrous (≥97% w/w) was obtained from Fisher scientific.

**Table 1:** The composition of the synthetic wastewater.

Component	Molecular Weight (g mol^-1^)	Concentration (mg L^-1^)	Total Carbon (mg L^-1^)	Company
D-Glucose anhydrous	180.16	1600	639.4	Fisher Scientific
Bacterial Peptone		480	227.8*	Fisher Scientific
Lab Lemco		320	144.9*	Oxoid
Ammonium Hydrogen Carbonate	79.06	160	24.3	Fisher Scientific
Potassium Hydrogen Carbonate	100.12	80	9.6	Fisher Scientific
Sodium Hydrogen Carbonate	84	80	11.4	Fisher Scientific

*measured by TC analysis.

### 2.2. Experimental procedure

**Fig 1:**
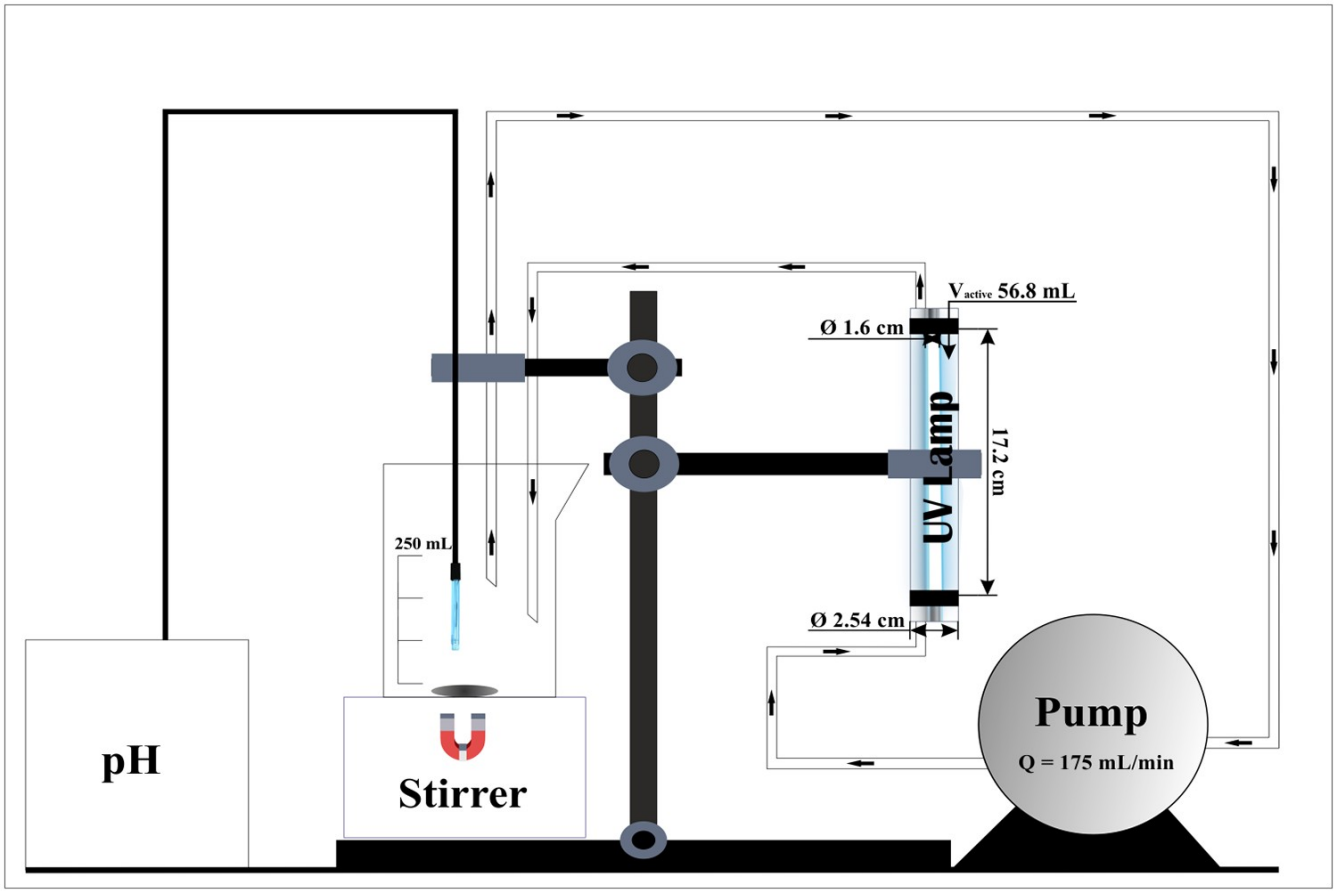
Experimental apparatus.

All experiments were conducted using an annular photoreactor operated in batch recycle mode with an OSRAM ultraviolet lamp with the following specifications: order reference HNS 6 W G5, voltage 42 V, nominal wattage 6.00 W, wavelength 254 nm, diameter 16.00 mm, length 212 mm, lifespan 9000 h. (Fig 1). The volume of the treated wastewater was 250 mL, whereas the irradiated volume was 56.8 mL. The non-irradiated part was continuously magnetically stirred. A peristaltic pump (Heidolph) was employed to achieve a 175 mL min^-1^ flow rate. All pH measurements were conducted by means of a LE409 electrode by Mettler Toledo.

Each experiment lasted 120 minutes and samples were withdrawn periodically for analysis. Prior to High Pressure Liquid Chromatography (HPLC) and Total Carbon (TC) analysis, samples were filtrated by means of Chromofil Xtra RC-20/25 filters (0.20 μm). TC concentrations were determined by means of a Multi N/C 3100 analyzer (Analytik Jena AG) and phenolic compounds were quantified using an Agilent 1290 Infinity HPLC. A summary of the experimental conditions is shown in Table 2. The details of HPLC and TC analysis are presented in Tables 3 and 4, respectively.

**Table 2:** Experimental conditions.

Parameter	Range	Comments
TC (mg L^-1^)	30–500	most experiments were performed with initial TC 32 mg L^-1^
Phenolic compound (mg L^-1^)	0/5/10	Ph or 2-CP or 2,4-DCP or 2,4,6-TCP or 4-NP was added keeping always initial TC equal to 32 mg L^-1^
TiO_2_ (g L^-1^)	0–1	most experiments were performed with 0.5 g L^-1^
H_2_O_2_ (mg L^-1^)	0–266	
Fe(III)	0/10	most experiments were conducted without iron addition
UV lamp (W / nm)	6 / 254	annular photoreactor
Total volume treated (mL)	250	irradiated volume 56.8 mL
Flow rate (mL min^-1^)	175	batch recycle mode
Experiment duration (min)	120	equal to irradiation time
Temperature (°C)	20–25	room temperature
Initial pH	6–7	no adjustment of pH

**Table 3:** The details of HPLC analysis.

Compound Parameter	Phenol	2-Chlorophenol	2,4-Dichlorophenol	2,4,6-Trichlorophenol	4-Nitrophenol
Column	ZORBAX Rapid Resolution High Definition column (Agilent), phase SB-C18, internal diameter = 2.1 mm, length = 100 mm, particle size = 1.8 μm				
Injection volume (μL)	5				
Eluent	50% ultra-pure water / 50% acetonitrile	85% ultra-pure water / 15% acetonitrile	85% ultra-pure water / 15% acetonitrile	85% ultra-pure water / 15% acetonitrile	85% ultra-pure water / 15% acetonitrile
Flow rate (mL min^-1^)	0.4				
Column temperature (°C)	20				
Detector wavelength (nm)	280				

**Table 4:** The details of TC analysis.

Carrier gas	Oxygen (99.999%)
Detector	Focus Radiation Non-Dispersive Infrared
Digestion method	high-temperature combustion
Combustion temperature (°C)	800
Catalyst	Platinum catalyst multi NC
Sample volume (μL)	250
Rinse volume (μL)	2000
Repetitions for TC	min 2, max 3. Coefficient of variation (CV) for TC ≤ 2%

The conversion of the phenolic compounds to intermediates and the TC removal because of CO_2_ production were calculated using the following formulas, respectively:

$$conversion\;(\%)=\frac{C_{0}-C_{t}}{C_{0}}\times 100$$ (1)

$$TC\;removal\;(\%)=\frac{[{TC]}_{0}-{\left[TC\right]}_{t}}{{\left[TC\right]}_{0}}\times 100$$ (2)

The average error was estimated to be below 3.5% (as one standard deviation of the mean) via repeating initially a few experiments in triplicates.

## 3 Results and discussion

### 3.1. UV/TiO_2_ process: The effect of initial TC and TiO_2_ concentration

Under UV light irradiation, electrons and positive holes are generated in the conduction (e^-^_cb_) and valence band (hv^+^_vb_) of titanium dioxide according to the first reaction [12]. The holes can either react directly with organic molecules (reaction 7) or form hydroxyl radicals (reactions 4–5) that subsequently oxidize organic molecules (reaction 8) [28,29]. The electrons can also react with organic compounds to provide reduction products (reaction 9). The role of oxygen (reaction 6) is important because it can react with the photo-generated electrons, preventing thus the fast recombination between electrons and holes on the catalytic surface [12].

$$TiO_{2}+h\nu \to e_{cb}^{-}+{hv}_{vb}^{+}$$ (3)

$${hv}_{vb}^{+}+{{OH}^{-}}_{surface}\to {OH}^{•}$$ (4)

$${hv}_{vb}^{+}+{H_{2}O}_{absorbed}\to \;OH^{•}+H^{+}$$ (5)

$$e_{cb}^{-}+{O_{2}}_{absorbed}\to O_{2}^{-•}$$ (6)

$${hv}_{vb}^{+}+Organic\;\to \;Oxidation\;products$$ (7)

$${OH}^{•}+Organic\;\to \;Degradation\;products$$ (8)

$$e_{cb}^{-}+Organic\to Reduction\;products$$ (9)

**Fig 2:**
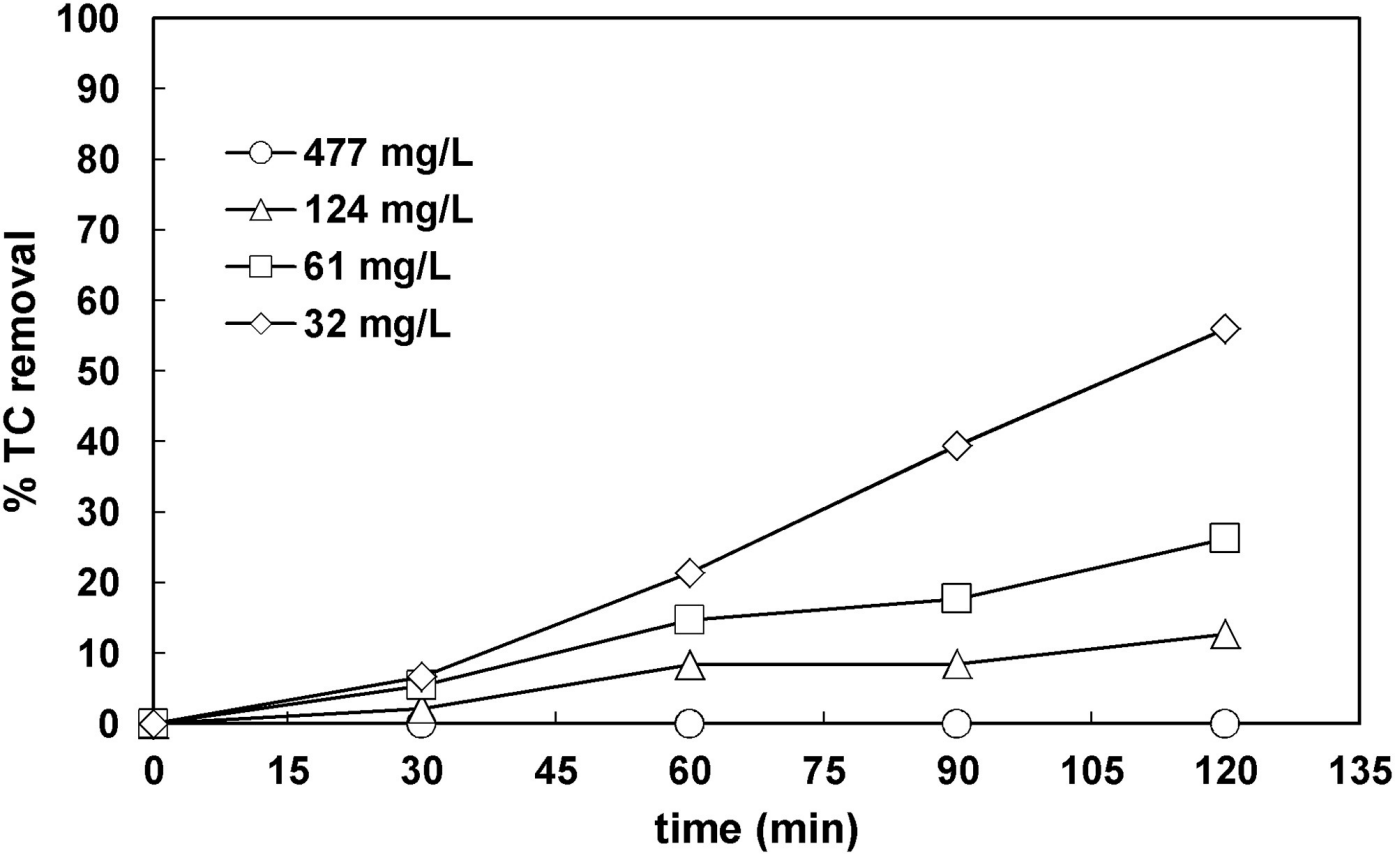
Effect of TC initial concentration on TC removal ([TiO_2_]_o_ = 1 g L^-1^).

Firstly, the impact of the initial total carbon on its removal was examined. The initial total carbon ranged in 30–500 mg L^-1^, whereas the concentration of TiO_2_ was 1 g L^-1^ for all the experiments shown in Fig 2. When the initial TC was above 120 mg L^-1^, the final TC removal observed was lower than 15%. The photocatalytic treatment was adversely affected by the initial carbon concentration due to the shortage of reactive species at increased concentrations [30]. Specifically, when the initial TC was increased from 32 to 61 mg L^-1^, the final removal of total carbon observed was decreased from 56% to 26%. Taking into account the final TC removal achieved, the initial TC concentration of 32 mg L^-1^ was used in the next experiments. TC concentrations close to this value have been reported previously for synthetic wastewaters effluents [26], and settled influent samples from wastewater treatment plants [31]. The photocatalytic treatment was adversely affected by the initial carbon concentration due to the shortage of reactive species at increased concentrations as expected [30].

**Fig 3:**
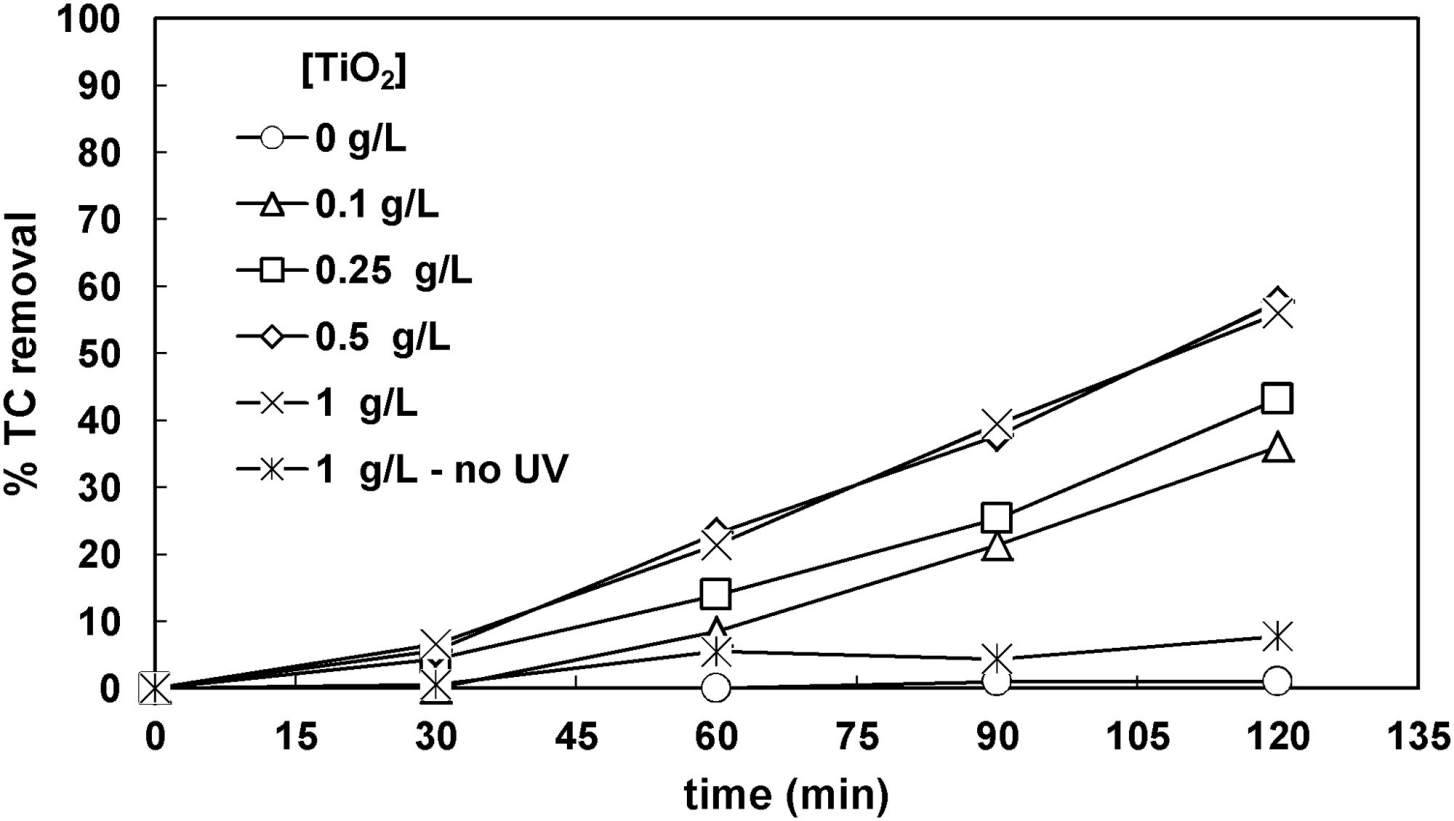
Effect of TiO_2_ loading on TC removal ([TC]_o_ = 32 mg L^-1^).

The effect of titanium dioxide loading ranging in 0.1-1g L^-1^ on TC removal was then studied for initial TC equal to 32 mg L^-1^. For 0.1 g L^-1^ TiO_2_, 36% TC removal was obtained. Increasing titanium dioxide loading to 0.5 g L^-1^ led to 58% TC removal, which remained practically the same when the TiO_2_ amount used was 1 g L^-1^, as shown in Fig 3. Increased concentrations of TiO_2_ above a certain value do not advance the process because UV irradiation cannot penetrate the treated solution [32]. It is also obvious that combining UV irradiation and photocatalyst was crucial for achieving considerable TC removals. Consequently, the concentration of 0.5 g L^-1^ was subsequently used.

For high initial concentrations of the target organic compound the oxidation rate can be increased significantly, whereas for moderate concentrations of the target compound, TiO_2_ concentration might not affect the process [33]. In contrast, for low concentrations of the target compound, higher oxidation rates can be observed for decreasing catalyst amounts. These observations are attributed to the trade-off between two competitive factors: the light penetration and the availability of active sites on catalyst surface; increased concentrations of TiO_2_ provide more active sites, however the light penetration is decreased and as a result the photo-irradiated part of the reactor is decreased too. The definition of the terms ‘‘low,” ‘‘medium,” and ‘‘high” for the initial concentration of the target compound depends on the photo-catalytic configuration.

### 3.2. UV/H_2_O_2_ process: The effect of H_2_O_2_ initial concentration

**Fig 4:**
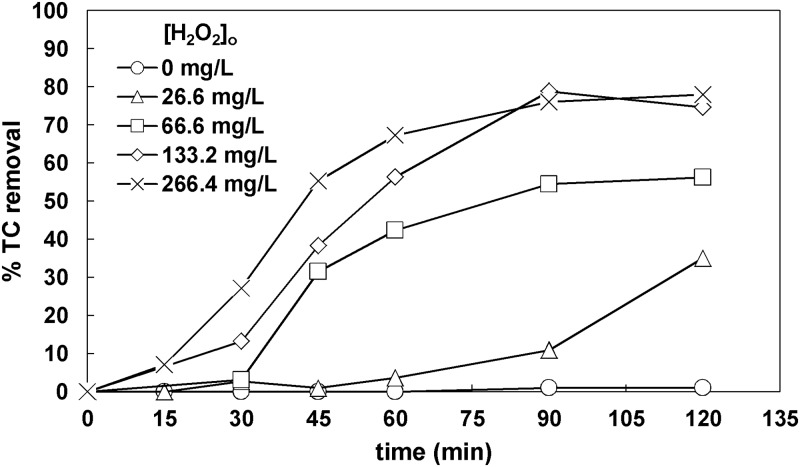
Effect of H_2_O_2_ initial concentration on TC removal ([TC]_o_ = 32 mg L^-1^).

For comparison reasons, the effect of H_2_O_2_ initial concentration in 27–266 mg L^-1^ was examined without TiO_2_. The initial total carbon was equal to 32 mg L^-1^. As it is shown in Fig 4, increasing concentrations of H_2_O_2_ up to 133.2 mg L^-1^ led to higher TC removals after 120 min. This can be explained by higher concentrations of OH• formed (reactions10-11); these radicals attack most organic compounds without discrimination [34].

$$H_{2}O_{2}⇄\;{HO}_{2}^{-}+H^{+}$$ (10)

$${HO}_{2}^{-}+H_{2}O\overset{hv}{\to }{2OH}^{•}+OH^{-}$$ (11)

Increasing the initial amount of H_2_O_2_ to 266 mg L^-1^ did not alter the final TC removal as previously observed [35–37], which can be attributed to the reaction of excess H_2_O_2_ with already produced hydroxyl radicals (reactions 12–13), namely H_2_O_2_ itself consumes the highly reactive OH• formed.

$$H_{2}O_{2}+•OH\;\to \;{HO}_{2}^{•}+H_{2}O$$ (12)

$${{HO}_{2}^{•}+•OH\to H}_{2}O+O_{2}$$ (13)

### 3.3. UV/TiO_2_/H_2_O_2_ process: The effect of combining TiO_2_ with H_2_O_2_

The effect of combining UV light, titanium dioxide as photocatalyst and hydrogen peroxide as oxidant on TC removal was subsequently studied. The initial concentrations used were: 32 mg L^-1^ total carbon, 0.5 g L^-1^ TiO_2_, and 66.6 mg L^-1^ H_2_O_2_. Combining TiO_2_ and H_2_O_2_ did not result in higher final TC removals.

There is no agreement in previously reported works about the effectiveness of combining hydrogen peroxide with titanium dioxide under UV irradiation. For example, on one hand it has been reported that such a combination increased the process efficiency as ultraviolet rays were coupled with both oxidant and photocatalyst [38], whereas on the other hand, competition for UV light between the oxidant and the photocatalyst [12] or the H_2_O_2_ adsorption on TiO_2_ that decreases catalyst activity [39] have been reported to decrease the overall process efficiency.

### 3.4. UV/TiO_2_/H_2_O_2_/Fe(III) process: The effect of ferric ions addition

The influence of ferric ions on the TC was also examined (photo Fenton-like process). Iron chloride (FeCl_3_, anhydrous) was added to produce ferric ions. The initial concentrations of TC, catalyst, oxidant and ferric ions along with the final TC removals achieved are presented in Table 5. The total carbon removal was increased from 52% for the UV/TiO_2_/H_2_O_2_ to 84% when Fe(III) was initially added. However, the presence of TiO_2_ was not beneficial as the same TC removal was observed after 120 min for both the UV/H_2_O_2_/Fe(III) and UV/TiO_2_/H_2_O_2_/Fe(III) processes. The explanation could be similar to the one stated in paragraph 3.1. The entirely different trend of the time curves obtained indicates that different kinetics were observed and that the oxidation of TC followed a different chemical reaction path for each process.

**Table 5:** The effect of Fe(III) presence on TC removal.

Reagents	UV/TiO_2_/H_2_O_2_	UV/H_2_O_2_/Fe(III)	UV/TiO_2_/H_2_O_2_/Fe(III)
TC (mg L^-1^)	32	32	32
TiO_2_ (g L^-1^)	0.5	0	0.5
H_2_O_2_ (mg L^-1^)	66.6	66.6	66.6
Fe(III) (ppm)	0	10	10
TC removal	52%	84%	84%

Photo Fenton is the easiest way to cause hydroxyl radicals formation without the necessity of using temperature, pressure or even UV light [40] although the reduction of Fe(III) to Fe(II) and oxidation of organics have been suggested to be greatly accelerated in the presence of ultraviolet irradiation [36,40]. The photo Fenton-like process starts using Fe(III) and reaction 14 instead of Fe(II) and reaction 15 in the case of the classic photo Fenton. Then, reactions 16 to 20 are common for both mechanisms.

$${Fe}^{3+}+H_{2}O_{2}\to {Fe}^{2+}+{HO}_{2}^{•}+H^{+}$$ (14)

$${Fe}^{2+}+H_{2}O_{2}\to {Fe}^{3+}+•OH+{OH}^{-}$$ (15)

$$•OH+H_{2}O_{2}\to {HO}_{2}^{•}+H_{2}O$$ (16)

$${•OH+Fe}^{2+}\to {Fe}^{3+}+{OH}^{-}$$ (17)

$${Fe}^{3+}+{HO}_{2}^{•}\to {Fe}^{2+}+O_{2}H^{+}$$ (18)

$${{Fe}^{2+}+{HO}_{2}^{•}+H^{+}\to Fe}^{3+}+H_{2}O_{2}$$ (19)

$${{HO}_{2}^{•}\to H}_{2}O_{2}+O_{2}$$ (20)

**Fig 5:**
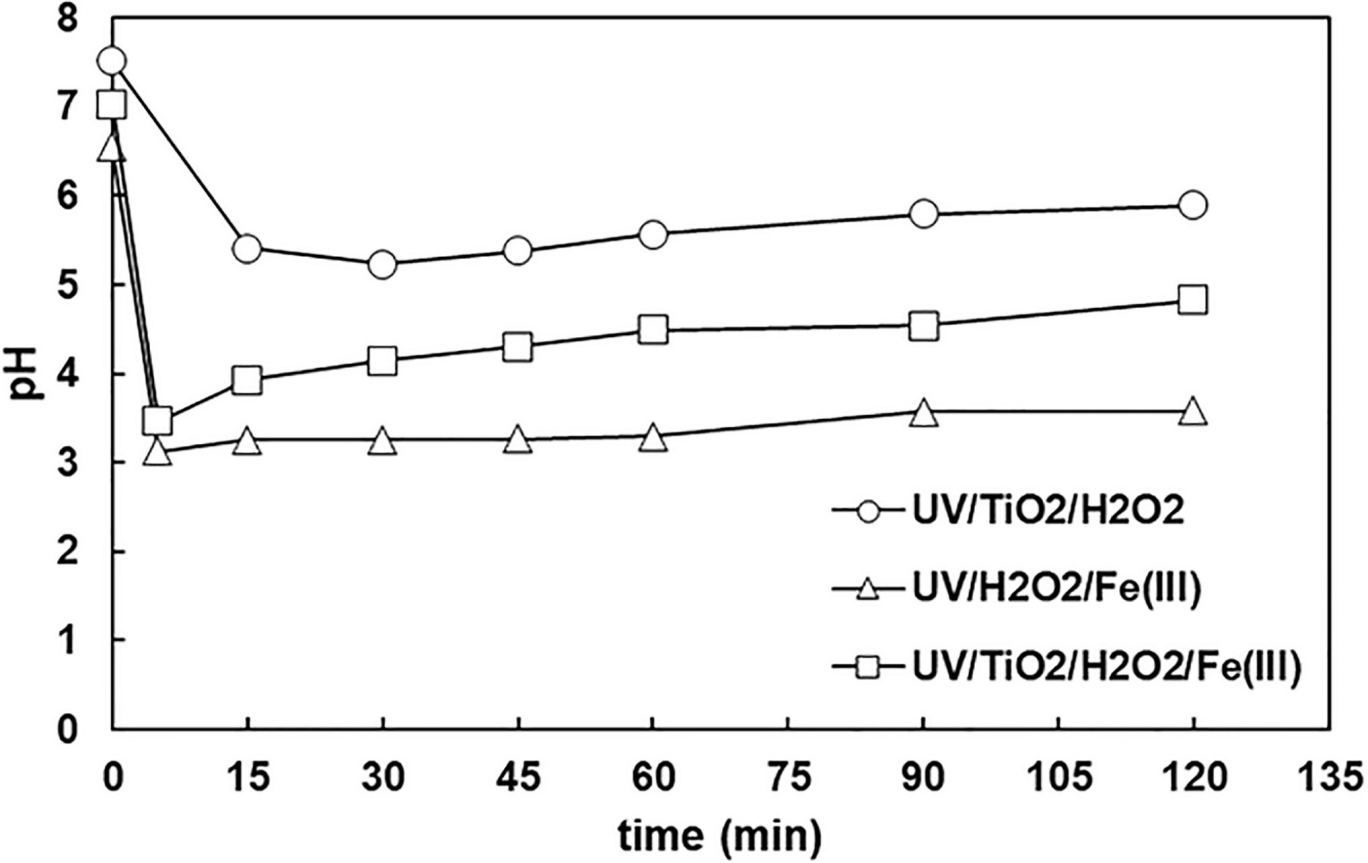
The evolution of pH with time ([TC]_o_ = 32 mg L^-1^, [TiO_2_]_o_ = 0.5 g L^-1^, [H_2_O_2_]_o_ = 66.6 mg L^-1^, [Fe(III)]_o_ = 10 ppm).

The pH evolution with time is presented in Fig 5. It started from a value around 7, then started to drop as the process proceeded, probably because of the transformation of organic carbon into organic acids. It has been reported that at the final step organic acids are slowly decomposed to CO_2_ leaving the solution, which results in increasing pH values [41].

**Fig 6:**
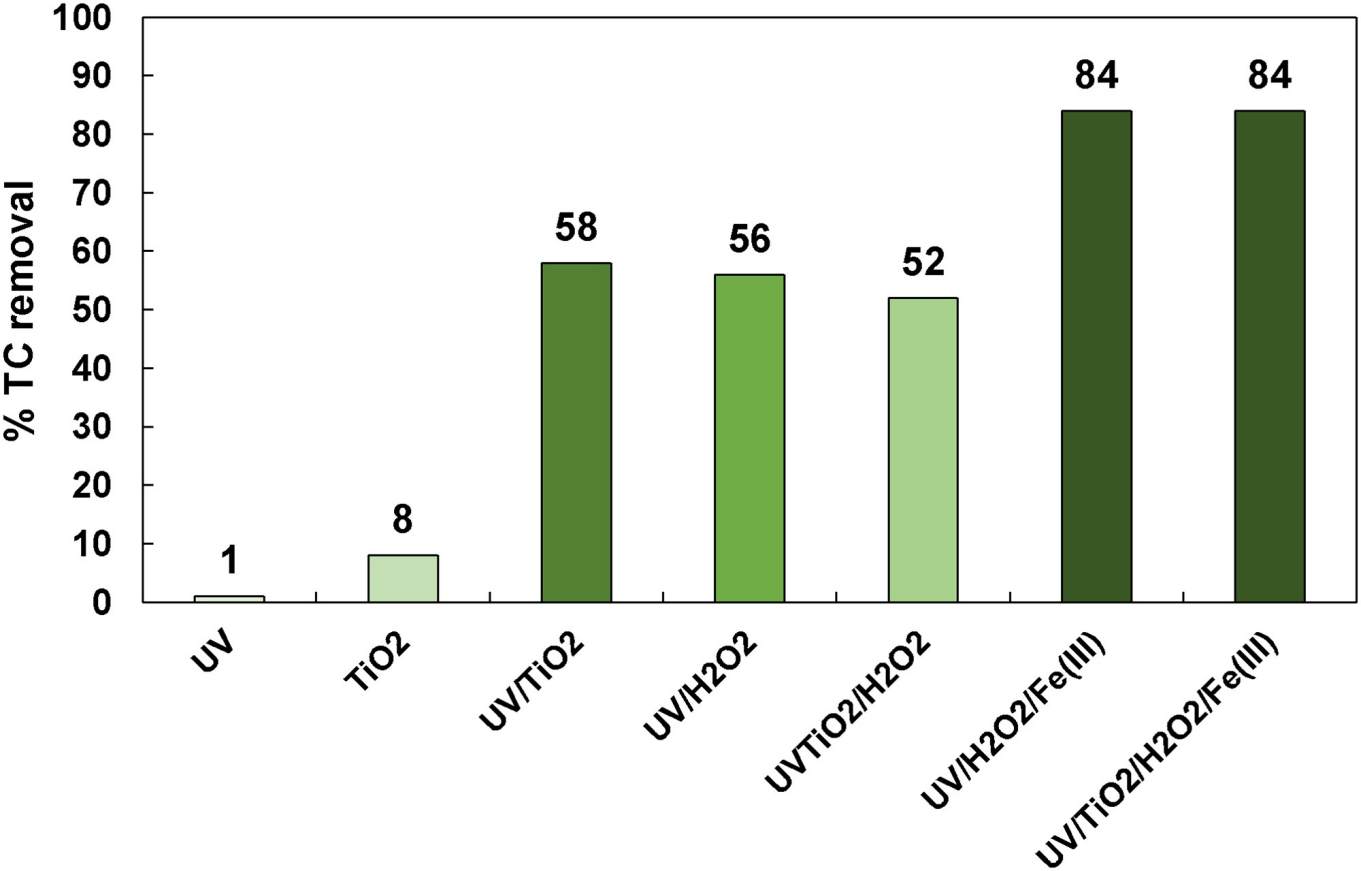
Final TC removal achieved in each process ([TC]_o_ = 32 mg L^-1^, [TiO_2_]_o_ = 0.5 g L^-1^, [H_2_O_2_]_o_ = 66.6 mg L^-1^, [Fe(III)]_o_ = 10 ppm).

In Fig 6, all TC removals obtained at the end of each process applied are shown.

### 3.5. Decomposition of phenolic compounds in wastewater

#### 3.5.1. Phenol

**Fig 7:**
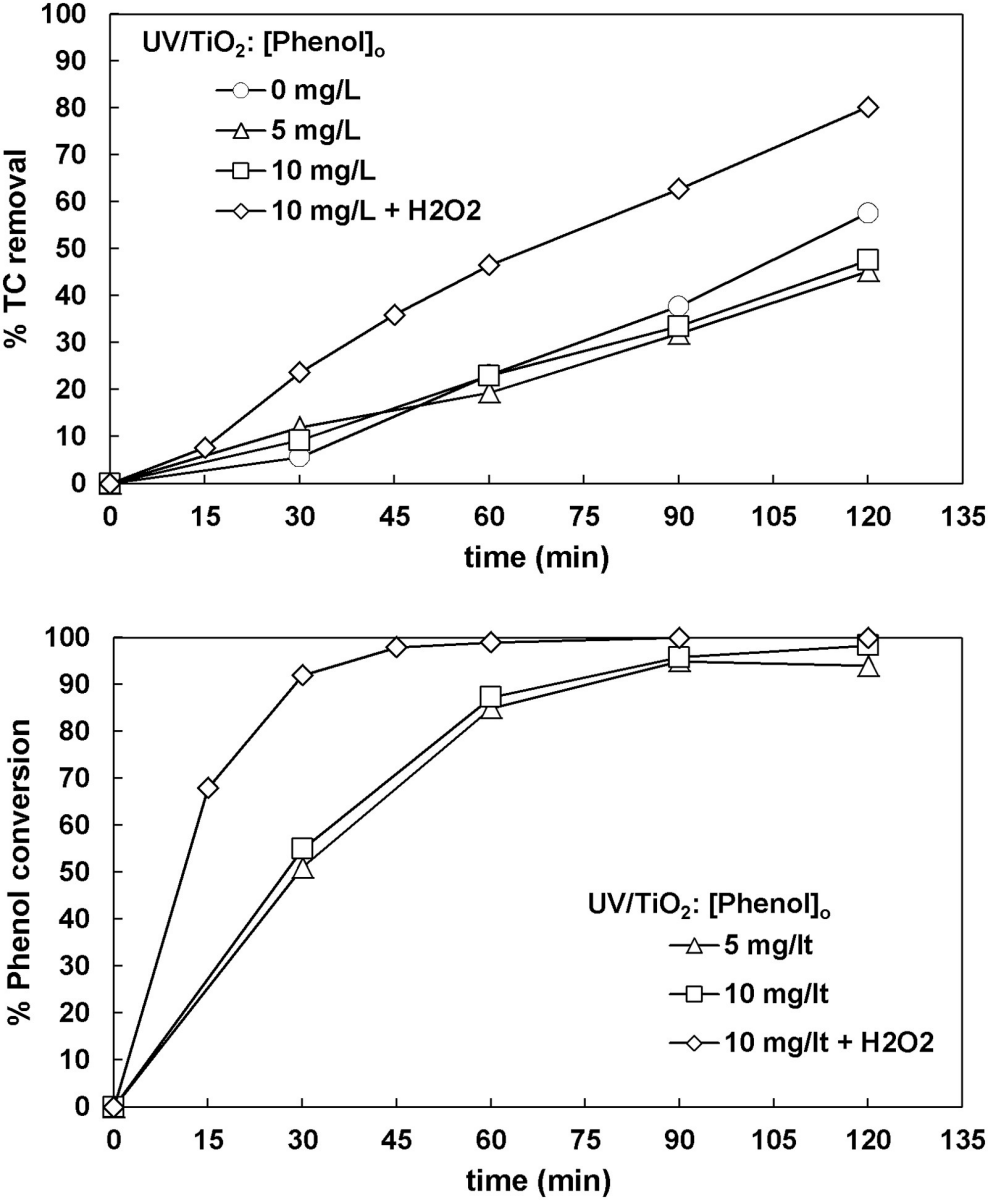
The effect of phenol concentration on TC removal and phenol conversion ([TC]_o_ = 32 mg L^-1^, [TiO_2_]_o_ = 0.5 g L^-1^, [H_2_O_2_]_o_ = 66.6 mg L^-1^ when used).

The photocatalytic process was also used to treat the synthetic wastewater with 5 mg L^-1^ or 10 mg L^-1^ of phenol. 0.5 g L^-1^ TiO_2_ was always used as photocatalyst, whereas one more experiment was conducted for 10 mg L^-1^ of phenol using additionally 66.6 mg L^-1^ H_2_O_2_. In all the experiments shown afterwards, the wastewater was always prepared so as to keep the same initial TC concentration of 32 mg L^-1^ as in the previous experiments. The results obtained are presented in Fig 7. The phenol conversion was practically the same for both initial phenol concentrations 5 and 10 mg L^-1^, whereas a higher conversion was achieved when H_2_O_2_ was used. Specifically, the TC removal was in the range of 45–48% in the presence of phenol, whereas 58% TC removal was achieved without phenol. The final phenol conversion was above 94% in all cases. From the results obtained, it can be suggested that phenol was rather easily decomposed by photocatalysis. However, it was not completely converted into carbon dioxide and water. Adding H_2_O_2_ seems to have provided surplus of hydroxyl radicals that promoted both phenol degradation and carbon mineralization, since the phenol conversion was 100% and the TC removal was 80% after 120 min.

#### 3.5.2. 2-Chlorophenol

**Fig 8:**
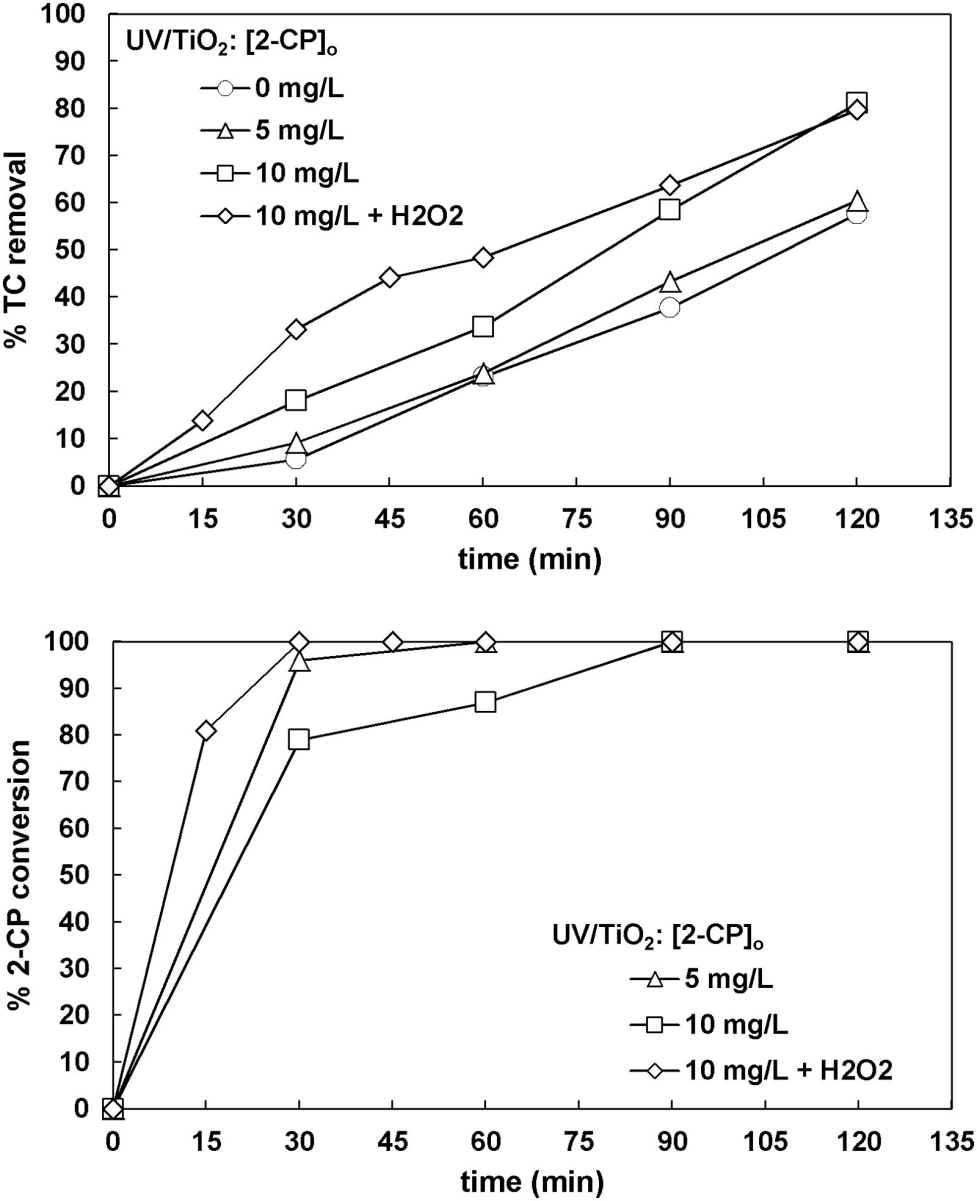
The effect of 2-chlorophenol concentration on TC removal and 2-chlorophenol conversion ([TC]_o_ = 32 mg L^-1^, [TiO_2_]_o_ = 0.5 g L^-1^, [H_2_O_2_]_o_ = 66.6 mg L^-1^ when used).

2-Chlorophenol (2-CP) was also added to the wastewater keeping the same initial TC. Increasing the initial chlorophenol concentration resulted in an increased final TC removal (Fig 8). The addition of H_2_O_2_ into the UV/TiO_2_ process promoted the mineralization of the wastewater during the first 90 min of treatment but did not result in any change in the final TC removal. In all cases, 2-CP was completely decomposed after 90 min.

#### 3.5.3. 2,4-Dichlorophenol

**Fig 9:**
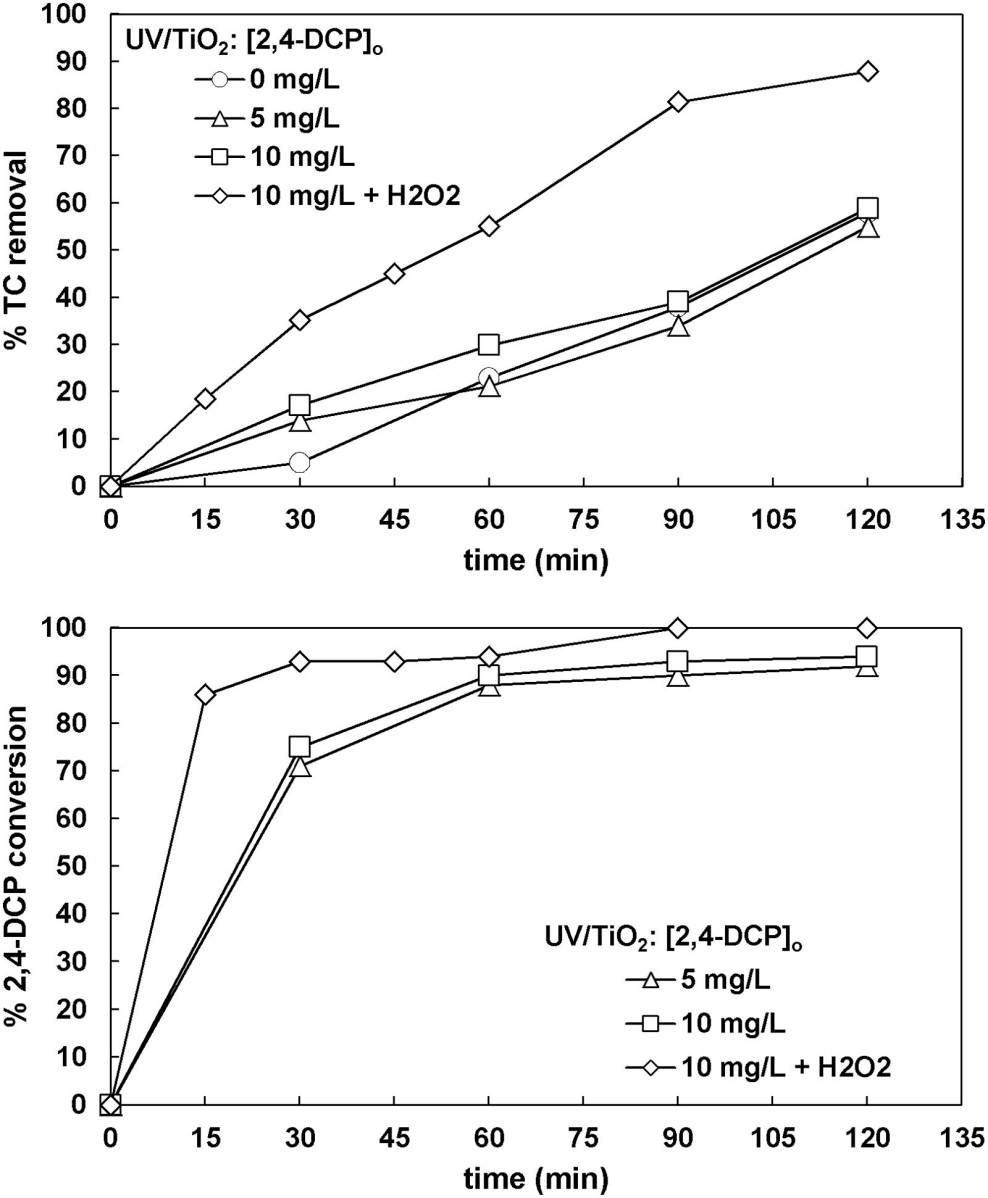
The effect of 2,4-dichlorophenol concentration on TC removal and 2,4-dichlorophenol conversion ([TC]_o_ = 32 mg L^-1^, [TiO_2_]_o_ = 0.5 g L^-1^, [H_2_O_2_]_o_ = 66.6 mg L^-1^ when used).

The results obtained for 2,4-dichlorophenol (2,4-DCP) are shown in Fig 9. 2,4-DCP had no effect on TC removal at the end of the process, whereas H_2_O_2_ was again beneficial for carbon mineralization. The conversion of 2,4-DCP was above 95% after 120 min.

#### 3.5.4. 2,4,6-Trichlorophenol

**Fig 10:**
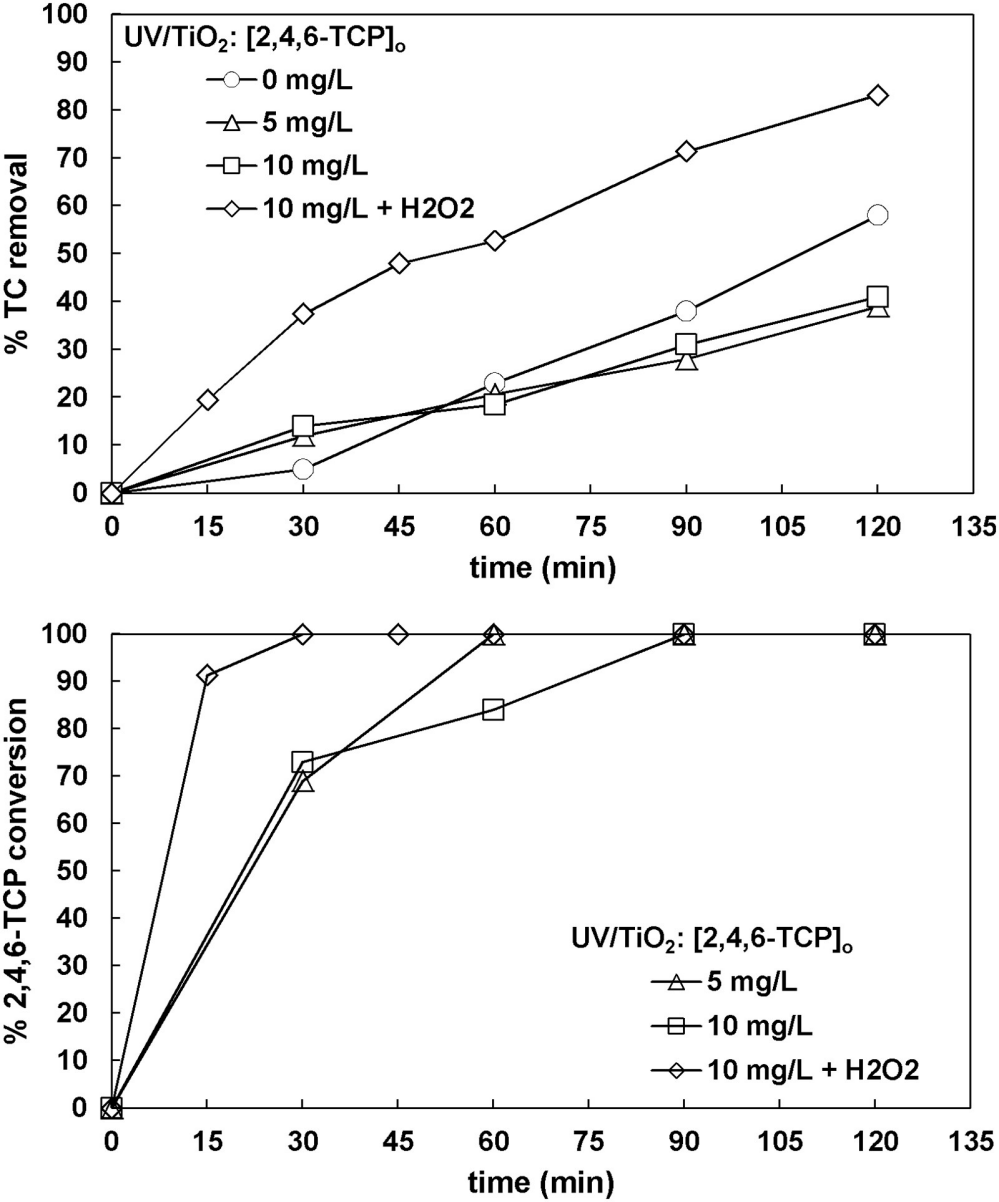
The effect of 2,4,6-trichlorophenol concentration on TC removal and 2,4,6-trichlorophenol conversion ([TC]_o_ = 32 mg L^-1^, [TiO_2_]_o_ = 0.5 g L^-1^, [H_2_O_2_]_o_ = 66.6 mg L^-1^ when used).

The results obtained for 2,4,6-Trichlorophenol (2,4,6-TCP) were similar to the ones obtained for phenol. There was no difference between 5 mg L^-1^ and 10 mg L^-1^ in terms of TC removal, whereas the presence of hydrogen peroxide enhanced the mineralization of the wastewater (Fig 10). According to HPLC results, the addition of H_2_O_2_ resulted in an enhancement of degradation rate, as 100% 2,4,6-TCP conversion was achieved in 30 minutes.

#### 3.5.5. 4-Nitrophenol

**Fig 11:**
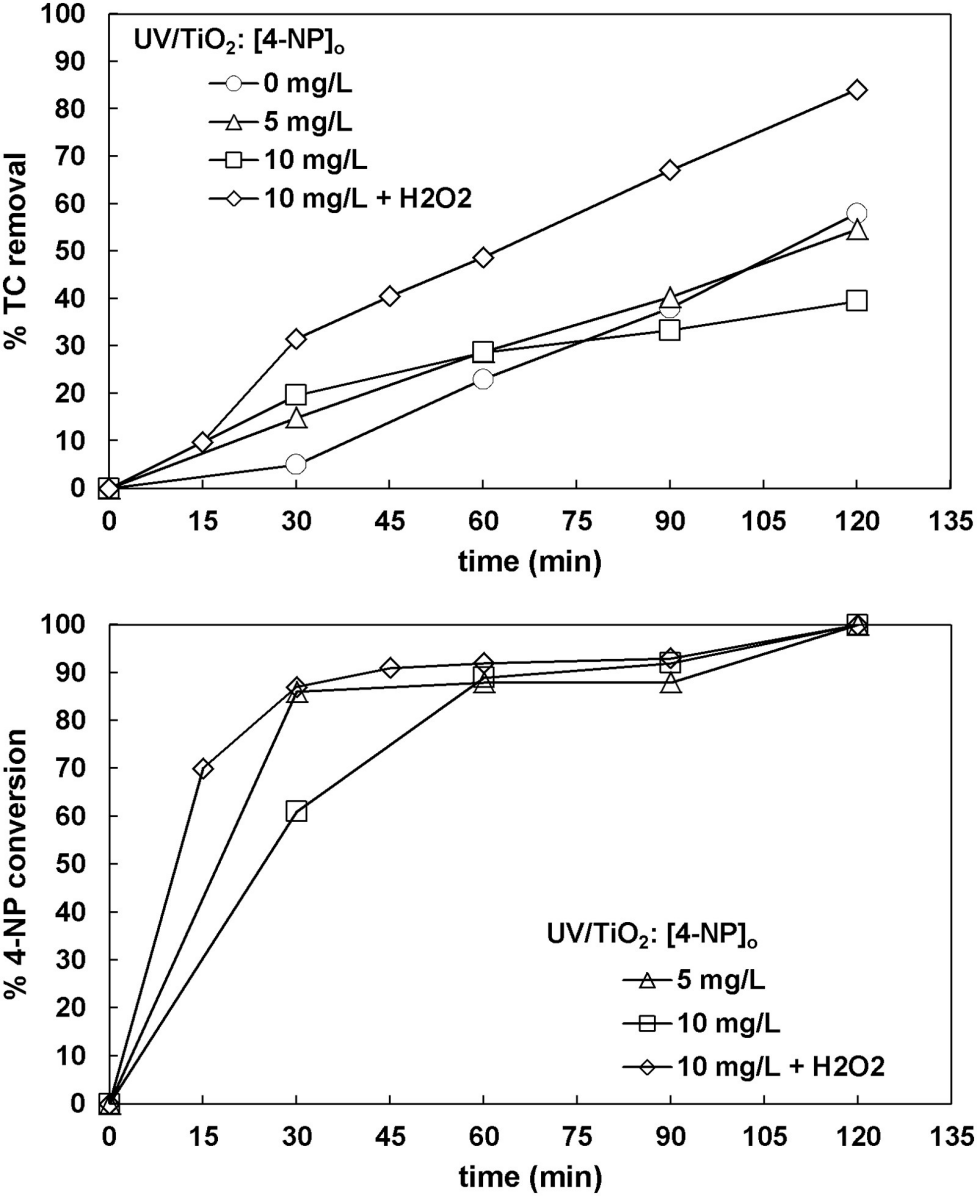
The effect of 4-nitrophenol concentration on TC removal and 4-nitrophenol conversion ([TC]_o_ = 32 mg L^-1^, [TiO_2_]_o_ = 0.5 g L^-1^, [H_2_O_2_]_o_ = 66.6 mg L^-1^ when used).

The results for TC removal in the case of 4-nitrophenol (4-NP) are shown in Fig 11. TC removals for 5 mg L^-1^ 4-NP were almost identical to the ones without any nitrophenol. In contrast, for 4-NP initial concentration equal to 10 mg L^-1^, the TC removal declined. The use of hydrogen peroxide was considerably beneficial. The conversion of 4-NP was complete in all cases after 120 min.

#### 3.5.6. Comparison

**Fig 12:**
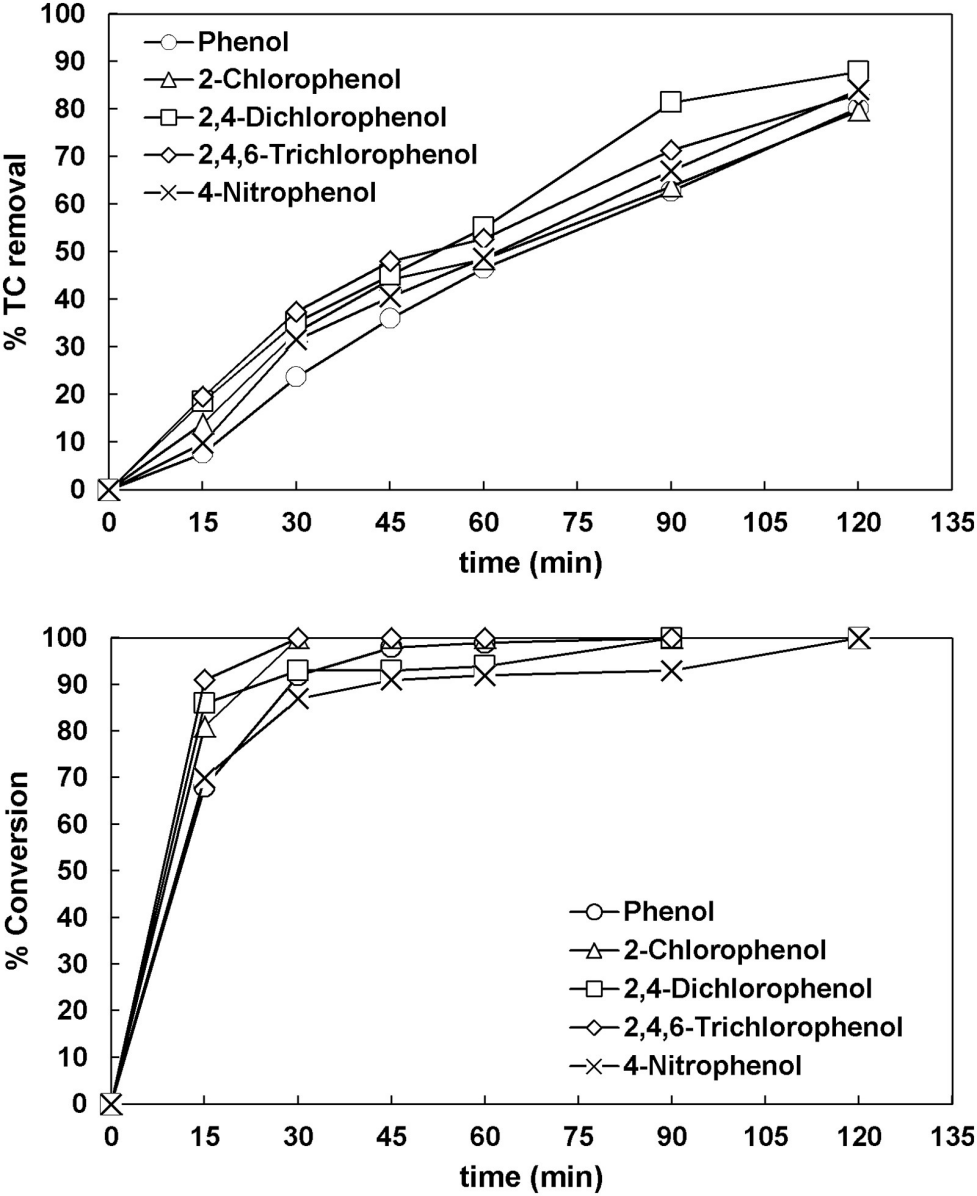
The effect of phenolic compound on TC removal and on conversion in the UV/TiO_2_/H_2_O_2_ process ([TC]_o_ = 32 mg L^-1^, [phenolic compound]_o_ = 10 mg L^-1^, [TiO_2_]_o_ = 0.5 g L^-1^, [H_2_O_2_]_o_ = 66.6 mg L^-1^).

All final TC removals observed in the treatment of synthetic wastewater containing phenolic compounds are shown in Table 6. In the UV/TiO_2_ process, the wastewater containing 2-CP was generally the easiest to mineralize whereas the one containing 2,4,6-TCP was the most resistant. The degradation order has been reported to be connected strongly to the photochemical system and the amounts of oxidant and catalyst initially used because they affect considerably the reaction pathway and thus the intermediates formed [12,42,43]. The addition of hydrogen peroxide eliminated the differences in TC removals observed from one compound to another at the end of the process as shown in Fig 12.

**Table 6:** TC removals after 120 min for wastewater containing phenolic compounds.

Concentration	Phenol	2-CP	2,4-DCP	2,4,6-TCP	4-NP
5 mg L^-1^	45%	60%	55%	38%	55%
10 mg L^-1^	48%	81%	59%	42%	39%
10 mg L^-1^ + H_2_O_2_	80%	80%	88%	83%	84%

It is clear that the photocatalytic treatment was able to effectively treat the synthetic wastewater containing concentrations at the ppm range of phenolic compounds. Especially, the UV/TiO_2_/H_2_O_2_ process removed more than 80% of total carbon in the wastewater and converted 100% all phenolic compounds after 120 min.

#### 3.5.7. Energy consumption

The knowledge of the electrical energy consumed by the UV lamp is important because it adds to the wastewater treatment operating cost. The electric energy per order, *E*_*EO*_ (kWh/m^3^/order), is commonly used, which can be estimated through the following equation for a batch reactor [44]:

$$E_{EO}=\frac{P∙t∙1000}{V∙60∙log(C_{o}/C_{f})}$$ (21)

where:

*P* = electrical power of the UV lamp, kW

*T* = irradiation time, min

*V* = the volume of the treated wastewater, L

*C*_*o*_ = the initial concentration of the pollutant, mg L^-1^

*C*_*f*_ = the final concentration of the pollutant, mg L^-1^

The values obtained are shown in Table 7. It is clear that the influence of the compound present in the wastewater affected substantially the energy consumed despite the fact that the total initial carbon was kept constant and it was only partially substituted. The extent of carbon substitution (5 or 10 mg L^-1^) affected *E*_*EO*_ too, whereas the addition of hydrogen peroxide led to lower energy consumptions and decreased significantly the differences from one compound to another. These observations are in accordance with previously reported ones. Specifically, Foteinis et al. [45] estimated *E*_*EO*_ values in the range of 4–958 kWh/m^3^/order in the photochemical oxidation of an endocrine disrupting micro-pollutant. The authors reported also the great dependence of energy consumption on the process applied and the fact that by adding small amounts of oxidative agents, the environmental footprint can be drastically decreased.

**Table 7:** The consumption of electrical energy (*E*_*EO*_, kWh/m^3^/order) per compound and treatment case.

Concentration	Phenol	2-CP	2,4-DCP	2,4,6-TCP	4-NP
5 mg L^-1^	185	121	216	248	138
10 mg L^-1^	169	67	124	191	224
10 mg L^-1^ + H_2_O_2_	69	69	52	62	60

## 4 Conclusions

In this work, the photocatalytic process was employed to treat a synthetic wastewater mainly composed of organic carbon. Various combinations of UV light, titanium dioxide, hydrogen peroxide and ferric ions were tested and the performance was evaluated in terms of carbon removal and target compound’s conversion. The main results are:

1. Both UV/TiO_2_ and UV/H_2_O_2_ processes were effective as TC removals 58% and 56% were achieved, respectively, for the following conditions: [TC]_o_ = 32 mg L^-1^, [TiO_2_]_o_ = 0.5 g L^-1^ and [H_2_O_2_]_o_ = 66.6 mg L^-1^. However, the combination of TiO_2_ with H_2_O_2_ resulted in only 52% TC removal.
2. The addition of ferric ions increased considerably the TC removal obtained (84%) for UV/H_2_O_2_ treatment. However, adding titanium dioxide as photocatalyst did not further improve the efficiency of the process.
3. When the carbon of the wastewater was partially substituted by either 5 mg L^-1^ or 10 mg L^-1^ of phenol or 2,4,6-trichlorophenol, the final TC removal in the UV/TiO_2_ process was decreased.
4. In the UV/TiO_2_ process, the wastewater containing 2-chlorophenol was generally the easiest to mineralize whereas the one containing 2,4,6-TCP was the most resistant. The addition of hydrogen peroxide eliminated the differences observed in relation with TC removal from one compound to another.
5. The consumption of electrical energy (*E*_*EO*_) estimated per compound and treatment case ranged in 52–248 kWh/m^3^/order. The addition of hydrogen peroxide in the UV/TiO_2_ eliminated the differences in calculated *E*_*EO*_ values from one compound to another.

## Supporting information

S1 File Total carbon removal, phenolic compound conversion and pH data for the photocatalytic treatment of organic pollutants in a synthetic wastewater using UV light and combinations of TiO_2_, H_2_O_2_ and Fe(III). (XLSX)
